# Antioxidant Status in the Vitreous of Eyes with Rhegmatogenous Retinal Detachment with and without Proliferative Vitreoretinopathy, Macular Hole and Epiretinal Membrane

**DOI:** 10.3390/life11050453

**Published:** 2021-05-19

**Authors:** Agata Pietras-Baczewska, Katarzyna Nowomiejska, Agnieszka Brzozowska, Mario Damiano Toro, Wojciech Załuska, Małgorzata Sztanke, Krzysztof Sztanke, Robert Rejdak

**Affiliations:** 1 Chair and Department of General and Pediatric Ophthalmology, Medical University of Lublin, 20-059 Lublin, Poland; katarzyna.nowomiejska@umlub.pl (K.N.); toro.mario@email.it (M.D.T.); robert.rejdak@umlub.pl (R.R.); 2 Department of Mathematics and Medical Biostatistics, Medical University of Lublin, 20-059 Lublin, Poland; agnieszkabrzozowska@umlub.pl; 3 Faculty of Medicine, Collegium Medicum Cardinal Stefan Wyszynski University, 01-815 Warsaw, Poland; 4 Department of Nephrology, Medical University of Lublin, 20-059 Lublin, Poland; wojciech.zaluska@umlub.pl; 5 Department of Medical Chemistry, Medical University of Lublin, 20-059 Lublin, Poland; malgorzatasztanke@umlub.pl; 6 Laboratory of Bioorganic Synthesis and Analysis, Chair and Department of Medical Chemistry, Medical University of Lublin, 20-059 Lublin, Poland; krzysztofsztanke@umlub.pl

**Keywords:** antioxidant status, vitreous body, rhegmatogenous retinal detachment, proliferative vitreoretinopathy

## Abstract

(1) Background: The aim of the study was to test the hypothesis that the antioxidant status in the vitreous body of eyes, which had been vitrectomized due to rhegmatogenous retinal detachment (RRD) with or without proliferative vitreoretinopathy (PVR), is higher than in eyes vitrectomized due to other retinal diseases. (2) Methods: four patient groups were analyzed: 22 eyes of patients with RRD without PVR, 27 eyes with RRD and PVR, 22 eyes with macular hole (MH) and 10 eyes with epiretinal membrane (ERM). Spectrophotometric methods were used to determine the total antioxidant status (TAS) values as well as superoxide dismutase (SOD) and glutathione reductase (GR) activities in the vitreous fluid samples. (3) Results: no significant differences in TAS values and antioxidant enzyme activities were observed among patient with RRD with and without PVR and with MH and ERM. The longer the duration of RRD leading to PVR and better postoperative visual acuity, the higher the TAS level. No significant differences were found between “macula on” and “macula off” subgroups within the RRD group and the RRD combined with PVR group. (4) Conclusions: The preliminary results do not support the thesis that the antioxidant status of vitrectomized eyes is different in patients with RRD with or without PVR in comparison to patients with MH and ERM. In patients with RRD, PVR presence and detached macula do not affect the values of TAS, SOD and GR in the vitreous fluid. The duration of the disease influences TAS in the vitreous in eyes with RRD complicated with PVR.

## 1. Introduction

Oxidative stress has been implicated in the development of retinal cellular damages in many retinal diseases [1], such as proliferative diabetic retinopathy (PDR) [2], age-related macular degeneration (AMD) [3] and retinitis pigmentosa [4]. Oxidative stress itself is caused by the imbalance between the production of reactive oxygen species (ROS) and the antioxidative defense system of the tissues. Reactive oxygen derivatives, such as superoxide anion radicals (O_2_^−∙^, hydrogen peroxide (H_2_O_2_), hydroxyl radical (^•^OH), peroxyl radical and singlet oxygen (^1^O_2_), are the most common radicals in the organisms. The most ROS are produced in the mitochondrial respiratory chain in both enzymatic and oxidation reactions [5]. Previous experimental studies had proven that photoreceptor cells themselves have the capacity of ROS production by NADPH oxidase in stress conditions [6].

Rhegmatogenous retinal detachment (RRD) is a sight-threatening condition with an incidence of approximately 1 out of 10,000 people [7]. RRD can be described as the separation of the neurosensory retina and the retinal pigment epithelium (RPE), secondary to subretinal fluid accumulation between these retinal layers. The separation of photoreceptors from the RPE causes photoreceptors’ oxygen and nutrient depravation, leading to ROS overproduction [8]. Proliferative vitreoretinopathy (PVR) is the major complication of RRD, with the occurrence estimated at 10% of all RDD cases [9]. Even though the exact etiology of PVR formation is not clear, it is supposed that it is associated with increased pro-inflammatory cytokines and growth factor concentrations in the vitreous fluid [10,11]. The current literature suggests that PVR is an abnormal wound healing response, associated with the intravitreal dispersion of retinal pigment epithelial cells or the breakdown of the blood–ocular barrier [12,13].

RRD is an indication for prompt surgical management, and the pars plana vitrectomy (PPV) is the most commonly chosen procedure [7,14]. The general aim of the technique is the removal of the vitreous body and the following retinal repair procedure [14]. Vitreous humor is an important ocular structure that plays a role in maintaining biochemical homeostasis, consuming molecular oxygen and protecting from oxidative damage [5,6,7,8,9,10,11,12,13,14,15,16,17]. Idiopathic macular holes (MH) are described as full-thickness retinal gaps in the neurosensory foveal area [18]. Although it is not a sight-threating disease, the visual acuity can be severely decreased [19]. Epiretinal membrane (ERM) is the accumulation of cells and the extracellular matrix-forming fibrocellular membrane on the vitreoretinal interface, and its etiology is idiopathic [20]. In these cases, PPV is extended with internal limiting membrane (ILM) peeling, gas endotamponade, and face-down postoperative positioning is the treatment of choice as in the cases of both MH and ERM [21,22].

Superoxide dismutase (SOD) and glutathione reductase (GR) are important antioxidant enzymes responsible for the scavenging free radicals [23].

Superoxide dismutase is an enzyme that catalyzes the two-stage dismutation reaction of superoxide anion radicals (O_2_^−∙^) into molecular oxygen (O_2_) and hydrogen peroxide (H_2_O_2_), using the redox properties of metals present in the enzyme’s active centers as a mechanism of catalytic activity [23]. This protects the cell not only against the action of superoxide anion radicals, but also indirectly prevents the formation of the most toxic hydroxyl radical in the reaction catalyzed by transition metal ions [23]. Depending on the location and presence of transition metal ions in the active center of the enzyme, three isoforms of superoxide dismutase have been identified in mammals {23]. Copper–zinc superoxide dismutase (Cu Zn-SOD and SOD-1) occurs in the cytoplasm and cell nucleus. In the active center, it contains copper atoms that perform a catalytic function and zinc atoms responsible for the stabilization of the enzyme molecule. It is assumed that 85–90% of the total SOD activity in the body depends on Cu and Zn-SOD. Manganese superoxide dismutase (Mn-SOD, SOD-2), present in the mitochondrial matrix, contains manganese atoms. Extracellular superoxide dismutase (EC-SOD) occurs in the interstitial spaces of tissues and in extracellular fluids (plasma, lymph, cerebrospinal fluid and synovial fluid), and contains copper and zinc ions in its active center [23].

Glutathione reductase (GR) occurs in the cytosol and mitochondria, and its main function is to maintain the proper concentration of glutathione in the cell by the regeneration of reduced glutathione (GSH) from glutathione disulfide (GSSG) with the participation of NADPH [24]. GSH is essential for the proper functioning of another important antioxidant enzyme, i.e., glutathione peroxidase (GPx). In addition, glutathione reductase protects proteins containing thiol groups against the effects of GSSG, which can form mixed disulfides with them or oxidize their thiol groups [24].

The aim of the study was to assess the antioxidant status, on the basis of total antioxidant status (TAS) values and antioxidant enzyme—SOD and GR—activities, in the vitreous fluid of patients with RRD, MH and ERM, to test the hypothesis that oxidative stress is increased in RRD, particularly with concomitant PVR.

## 2. Materials and Methods

The study was designed as a prospective observational study. The study protocol was approved by the Ethics Committee of Medical University of Lublin, Poland (number of approval KE-0254/56/2020). Written informed consent was taken from each participant after explaining the nature of the study. The study was conducted at the Department of General Ophthalmology and Pediatric Ophthalmology Service, Medical University of Lublin, Poland, between the years 2018 and 2020. The study group consisted of 73 consecutive patients (39 women and 34 men), who underwent vitrectomy and fulfilled the inclusion criteria (Table 1). One eye of each patient was included in the study. Inclusion criteria were as follows: symptoms and signs of RRD (e.g., decreased central visual acuity, visual field loss, floaters and detached retina in fundoscopy) confirmed with B-scan ultrasonography; exclusion criteria were as follows: prior vitrectomies, diabetes mellitus, vitreous hemorrhage, glaucoma and uveitis. The age average was 68.27+/−13.29 years (range from 12 to 89 years). Patients were subdivided into four groups: patients suffering from RRD with PVR (n = 27; mean age: 64 years; 10 females; 17 males), RRD without PVR (n = 22; mean age: 67 years; 10 females; 12 males), ERM (n = 10; mean age: 73 years; 7 females; 3 males) and MH (n = 14; mean age: 72 years; 12 females; 2 males). Eyes with ERM and MH served as the control group. There were no significant differences in regard to age between the patient groups (H = 4.46, p = 0.22). The majority of the patients (n = 49) suffered from RRD, which represents 67.12% of the study group. All eyes were pseudophakic. The group of patients suffering from RRD was divided into patients with attached macula (“macula on”) as a beginning stage and detached macula (“macula off”) as an advanced stage of RRD. 

Each patient was examined with a slit lamp, patients with RRD had additional B-mode ultrasound examination, while MH and ERM were examined with optical coherence tomography (OCT) of the macula to confirm the diagnosis. Best corrected vision acuity was examined with use of the Snellen charts before the surgery and two week after the surgical intervention All the patients with RRD and PVR had grade C PVR according to the classification of “Retina Society Terminology Committee (1983)” [25] and updated Retina Society Classification (1991) [26], which means marked PVR with full-thickness fixed retinal folds and the presence of preretinal or subretinal membranes. Stages A and B were not included as they are more subclinical. Grade A (minimal PVR) is limited to the presence of vitreous cells or haze. Grade B (moderate PVR) is defined by the presence of rolled or irregular edges of a tear or inner retinal surface wrinkling, denoting subclinical contraction. 

Each surgery was performed by the same surgeon (KN). Undiluted vitreous fluid was acquired from the vitreous cavity at the beginning of PPV in all procedures. The vitrector line was connected to a sterile syringe (5 mL). After the closure of the aspiration line, undiluted core vitreous (500–1000 µL) was aspirated into the syringe by active vitrector cutting and syringe suction (Constellation; Alcon Instruments, Fort Worth, TX, USA). All vitreous samples were snap-frozen and then stored at −80 °C until assay.

All the samples were submitted to the Department of Medical Chemistry, Medical University of Lublin, Poland, in order to determine the antioxidant parameters. The values of total antioxidant status and activities of antioxidant enzymes, i.e., superoxide dismutase and glutathione reductase, were measured in the vitreous fluid. TAS, SOD and GR were assayed with RANDOX kits according to the manufacturer’s instructions. All determinations were performed two or three times.

TAS was determined spectrophotometrically using the ready-to-use diagnostic kit TAS (Randox Laboratories Ltd., Crumlin, UK). In this method, 2,2′-azino-di-(3-ethylbenzothiazoline-sulphonate) (ABTS®) incubated with a peroxidase (metmyoglobin) and hydrogen peroxide forms a relatively stable blue-green radical cation (ABTS). The low molecular weight antioxidants present in the added sample inhibit the formation of this colored product in proportion to their concentration. The color intensity of ABTS^®•+^ is measured at 600 nm. Briefly, 20 μL of sample (the vitreous fluid), standard (6-hydroxy-2,5,7,8-tetramethylchroman-2-carboxylic acid) or double deionized water (blank) was added to 1000 μL of the freshly prepared chromogen (metmyoglobin, 6.1 μmol/L; ABTS^®^, 610 μmol/L) in phosphate buffered saline (80 mmol/L, pH 7.4). After mixing and pre-warming to 37 °C, the initial absorbance of the sample/standard/blank was measured. Next, 200 μL of substrate (hydrogen peroxide, 250 μmol/L) was added, and after 3 min, the final absorbance was measured. Results are expressed in mmol of TAS/g of protein.

The activity of superoxide dismutase (SOD) was determined spectrophotometrically using the ready-to-use diagnostic kit RANSOD (Randox Laboratories Ltd., Crumlin, UK). In this method, the superoxide anion radical (O_2_^•-^), produced from xanthine in the reaction catalyzed by xanthine oxidase, reacts with 2-(4-iodophenyl)-3-(4-nitrophenol)-5-phenyltetrazolium chloride, resulting in the formation of a red formazan dye. The superoxide dismutase present in the added sample inhibits this reaction by converting the superoxide anion radicals into hydrogen peroxide and molecular oxygen. The color intensity of a red formazan dye measured at 505 nm is proportional to the SOD activity. Briefly, 30 μL of sample (the vitreous fluid) or standard (0.01 mol/L phosphate buffer pH 7.0 in redistilled water) was added to 1000 μL of the freshly prepared substrate (xanthine, 0.05 mmol/L; I.N.T., 0.025 mmol/L) in buffer (CAPS, 40 mmol/L, pH 10.2; EDTA, 0.94 mmol/L). After mixing and pre-warming to 37 °C, 150 μL of xanthine oxidase (80 U/L) was added. The initial absorbance was recorded after 30 s, and then, the absorbance was measured for a further 3 min. Results are expressed in U of SOD/mg of protein. 

The activity of glutathione reductase (GR) was determined spectrophotometrically using the ready-to-use diagnostic kit GLUT RED (Randox Laboratories Ltd., Crumlin, UK). The assessment of GR activity is based on the ability of glutathione reductase to catalyze the reduction of glutathione disulfide (GSSG) to glutathione (GSH) with a concomitant oxidation of NADPH to NADP^+^. This process is accompanied by a decrease in absorbance at 340 nm. Briefly, 40 μL of sample (the vitreous fluid) was mixed with 1000 μL of the freshly prepared substrate (GSSG, 2.2 mmol/L) in buffer (potassium phosphate, 250 mmol/L, pH 7.3; EDTA, 0.5 mmol/L). Next, 200 μL of NADPH (0.17 mmol/L) was added. The absorbance of the sample was measured every 1 min for 5 min. Results are expressed in U of GR/g of protein. 

The concentration of protein was determined by the Bradford method [27] using bovine serum albumin (BSA) as the standard. All the assays were performed using a Hitachi U2800 spectrophotometer (Hitachi, Tokyo, Japan). 

### Statistical Analysis

All calculations were performed using STATISTICA 13.0 (StatSoft, Krakow, Poland) software. The values of the measurable parameters analyzed were presented using the mean value, median, quartiles, and standard deviation, and for the unmeasurable ones using the number and percentage. For measurable features, the normality of the distribution of the analyzed parameters was evaluated using the W Shapiro–Wilk test. 

A nonparametric statistical Wilcoxon signed-rank test was used to compare dependent variables of two related samples. A nonparametric test, the Mann–Whitney U test, was used to compare independent variables in two groups. Patient age was compared with the use of the he Kruskal–Wallis test. Spearman’s rank correlation coefficient was used to assess the relationship between the variables. 

A p value ≤ 0.05 was considered statistically significant. 

## 3. Results

**Figure 1:**
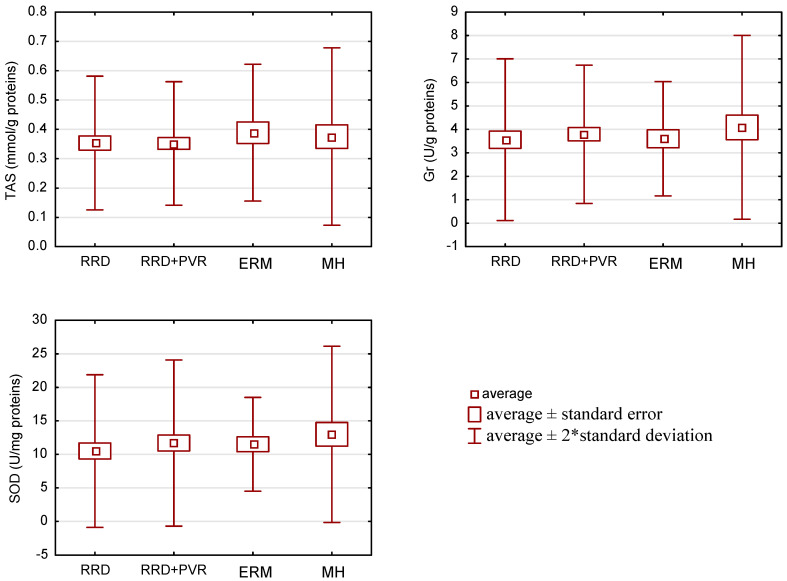
Antioxidant status [total antioxidant status (TAS) values as well as superoxide dismutase (SOD) and glutathione reductase (GR) activities in patients with rhegmatogenous retinal detachment (RRD) with and without PVR, epiretinal membrane (ERM) and macular hole (MH)).

No statistical differences between groups in TAS values (p = 0.81) were found. However, TAS levels were the highest in the ERM and MH groups (Table 2, Figure 1).

SOD activity was the highest in the MH group (12.989 U/mg protein), while the differences in the activity of this enzyme between the groups were not statistically significant (p = 0.67) (Table 3; Figure 1).

Additionally, the activity of GR was slightly higher in the MH group in comparison to the RRD, RRD with PVR and ERM groups. These differences were not statistically significant (p = 0.80) (Table 4; Figure 1).

The analysis showed that the disease duration was significantly longer in the ERM with MH groups, compared to RRD and RRD combined with the PVR group (p = 0.0001), (Table 5).

The analysis showed a significant correlation between the disease duration and the TAS level in the PVR group between the duration of the disease and the TAS activity level (R = 0.48). The longer the duration of the disease leading to PPV, the higher the TAS level. There was no significant difference between the duration and the SOD and GR activity levels in the PVR and the RRD groups (p > 0.05) (Table 6).

There was no correlation found between the duration of the disease and antioxidant levels in the MH and ERM groups.

Median postoperative visual acuity was 0.3 Snellen (0.09–0.4) in the group with RRD, 0.1 Snellen (0.06–0.3) in the group with RRD and PVR, 0.2 Snellen (0.1–0.4) in the group with ERM and 0.3 Snellen in the group with MH (H = 8.53, p = 0.04). There was a significant correlation between postoperative visual acuity and TAS levels in the group with PVR (R = −0.39, p = 0.04).

“Macula off” was present in 72% of eyes with PVR and in 50% of eyes without PVR. Statistical analysis showed no significant differences (p > 0.05) between “macula on” and “macula off” subgroups in both groups in regard to TAS, SOD and GR.

## 4. Discussion

The present study did not support the hypothesis that oxidative stress is increased in the vitreous body of eyes vitrectomized due to RRD and PVR. However, it may expand the insight into the pathophysiology of RRD, which is very important as the incidence of RRD is increasing [28]. We showed that a longer duration of RRD leading to PVR is correlated with a higher TAS level in the vitreous.

It has already been proven that high oxidative stress damages the retina, by the acceleration of photoreceptors’ and ganglion cells’ apoptosis. Most authors describe the influence of increased oxidative stress in the diabetic retinopathy and age-related macular degeneration. Recent investigations provided the information that metabolic processes in the pathological retina increase oxidative stress and lead to PVR formation [29]. In this study, the total antioxidant status (TAS) as well as two antioxidative enzymes, i.e., superoxide dismutase (SOD) and glutathione reductase (GR), were chosen to assess the antioxidant status of the vitreous fluid in retinal diseases.

There are many methods of oxidative stress evaluation. In biological samples, oxidative status is assessed by direct ROS level measurement, biomolecule damage detection (lipids, DNA and proteins) or antioxidative status determination (enzymatic or nonenzymatic antioxidant levels) [30]. As recommended, enzyme activities were evaluated together with total antioxidant status to exclude possible interactions between particular agents. The TAS values and antioxidant enzymes activities reflect the antioxidant status of the tissues, while deceased levels of the enzymatic and nonenzymatic antioxidants indicated increased oxidative stress. A longer RRD duration leads to higher TAS in the vitreous, which we can conclude on the basis of the results of the present study.

Maeno and colleagues reported increased oxidative stress in vitreous fluid in patients with RRD [9]. The studies compared biological antioxidant potential (BAP) in the vitreous fluid collected from patients with RRD, proliferative diabetic retinopathy (PDR), retinal vein occlusion, ERM and MH. The BAP values were determined by measuring the reducing potential of the Fe^3+^ to Fe^2+^ conversion in thiocyanate solution. Significantly increased oxidative stress was present in RRD compared to MH; only the extent of the detachment was significantly correlated with the BAP, with no significant relationships found for the duration, presence of PVR or VH, macular status or patient age. RRD exhibited a significantly lower BAP value than MH, while proliferative diabetic retinopathy (PDR) had a significantly smaller BAP than ERM and MH. However, there were no statistically significant differences in the BAP values among RRD, PDR, RVO and ERM. This is consistent with our results; however, different parameters of antioxidant status were assessed. Maeno et al. reported the outcomes of relatively small patient groups. Furthermore, the average age of the patients was statistically different. However, the authors detected no significant correlation between the BAP and the patients’ age, suggesting that the patient age is not likely to influence the BAP values in the disorders studied.

Moreover, Maeno and colleagues reported that RRD itself had a much greater influence on vitreal oxidative stress than free hemoglobin in vitreal hemorrhage, previously described as the ROS production agent.

Cederlund and colleagues as well as Takahashi and co-workers reported the correlation of oxidative stress with RRD severity 8311. Both teams expressed the severity of RRD in the presence or absence of vitreal haemorrhage, RRD extent (quadrants) and macular status.

In the study of Cederlund and colleagues [31], levels of carbonyl groups, a marker of oxidative stress, and α(1)-microglobulin (A1M) were measured in 14 vitreous samples derived from patients suffering from RRD, and compared with 14 samples from macular hole patients. Levels of carbonyl groups and A1M varied widely in RRD vitreous samples, but were significantly higher in samples derived from eyes with a large detachment area and macula-off status. Moreover, RRD samples displayed significantly higher levels of A1M, whereas changes in total protein levels and carbonyl groups were not significant. Authors concluded that oxidative stress is a prominent feature of human eyes with primary RRD, and is directly related to detachment severity; however, the study groups were very small.

In the present study, we categorized eyes vitrectomized due to RRD as without PVR and with PVR C, which means longer lasting RRD with a larger extent of RRD and with “macula off”. According to the definition of PVR, which is a complication of RRD [25], it can be assumed that the RRD with PVR is more severe. Most authors refer only to the most advanced stages of PVR, basically, grade C. The early stages of PVR (named as grades A and B), which are common to all classifications, are not used by clinicians [32].The classifications of PVR are, to date, purely descriptive and do not reflect the pathobiology of this complex vitreoretinal disease. Verdejo and colleagues [33] showed that fibrovascular proliferative vitreoretinopathies correlate with increased free radical formation and decreased antioxidant activity in the human vitreous body. Our results showed no differences in TAS values and evaluated enzymes activities in patients with RRD with or without PVR.

Takahashi and co-workers also reported no relationship among the RRD duration, macular status, PVR or HV presence or patients’ age [8]. In a study analyzing the metabolomics of human vitreous in diabetic retinopathy and rhegmatogenous retinal detachment, [34] profound changes were observed in diabetic retinopathy vitreous, including altered glucose metabolism and the activation of the pentose phosphate pathway, which provides reducing equivalents to counter oxidative stress. In contrast, the vitreous metabolite profiles of retinal detachment patients were similar to the controls. In our study, we did not measure antioxidant levels in diabetic retinopathy, but we did not observe significant differences between retinal detachment and the control, which consisted of eyes with MH and ERM.

Wert and associates conducted a study assessing the effect of SOD3 on oxidative stress in the vitreoretinal interface. The study was carried out on both a human and a mouse vitreous model. The results shown that the regulation of SOD3 at the vitreous base and cortex may represent a future therapeutic target for vitreoretinal diseases. Material collected during the research from vitreous bodies of humans and mice showed higher levels of oxidative stress biomarkers (nitrates and/or 3-nitrotyrosine) suggesting SOD3 dysfunction. The dysfunction or complete lack of SOD3, and thus increased oxidative stress, causes internal damage to the inner retina, which can be part of DVR pathogenesis and other vitreoretinal diseases [35]. In our study, the SOD activities did not differ statistically in the vitreous of patients with RRD with or without PVR considered for the most severe of all the studied diseases.

Mancino and colleagues investigated total antioxidant capacity (TAC) in the blood, aqueous humor, and vitreous bodies of diabetic and nondiabetic patients [36]. The total antioxidant capacity was determined with the oxygen radical absorbance capacity method and according to energy charge potential. PDR patients had lower levels of TAC in the vitreous and aqueous humor, but not in the blood, compared with nonproliferative diabetic retinopathy patients. These results suggest that the development of diabetic retinopathy is associated with high-level oxidative stress and diminished antioxidant defenses [36,37]. This report encourages the further investigation of the vitreous body. Comorbidities and coexisting conditions, especially diabetes, should be additional variables. In these cases, other body fluids oxidative status could be compared to vitreous humor. In the present study, there were no patients with diabetes included.

We did not find any age dependence of antioxidants, and there were no significant differences in age between groups. The similar findings were reported by Brzović-Šarić and colleagues [38], who analyzed different oxidative stress markers in the vitreous and serum of diabetic retinopathy patients and also MH, RRD and ERM.

The limiting factor for our study and its control group is that no vitreous of healthy subjects was included, as there were no indications for PPV in healthy subjects, and they were not performed. Thus, as a control group, MH and ERM eyes were included in the study, as conducted in previous studies [9,31,39]. Both MH and ERM are degenerative diseases of the vitreoretinal interface, with a long duration, longer than RRD; thus, they may also lead to increased TAS, SOD and GR in the vitreous. Further studies with a control group consisting of the vitreous of normal eyes might show differences in TAS, SOD and GR levels in comparison to RRD, MH and ERM.

New therapies decreasing harmful ROS formation will be the step forward in oxidative stress-derived ophthalmic diseases. Currently, there are only cell or animal model studies that demonstrate the beneficial use of antioxidants in the prevention of retinal damage. The treatment course may aim to both reduce ROS and promote the production of antioxidant enzymes.

## 5. Conclusions

A longer duration of RRD leading to PVR is correlated with a higher TAS level in the vitreous.

## Figures and Tables

**Table 1:** Patients’ characteristics (RRD—rhegmatogenous retinal detachment; PVR—proliferative vitreoretinopathy; ERM—epiretinal membrane; MH—macular hole; BCVA—best corrected vision acuity).

Diagnosis	Size of the Group (Number of Patients)	Gender (Females/Males Ratio)	Mean Age (years)	BCVA before Surgery (Snellen)	BCVA after Surgery (Snellen)
RRD without PVR	22	10F/12M	67	0.10	0.30
RRD with PVR C	27	10F/17M	64	0.05	0.10
ERM	10	7F/3M	73	0.20	0.20
MH	14	12F/2M	72	0.10	0.30

**Table 2:** Total antioxidant status (TAS) (mmol/g protein) values in the studied groups (RRD—rhegmatogenous retinal detachment; PVR—proliferative vitreoretinopathy; ERM—epiretinal membrane; MH—macular hole).

Group	Average	Standard Deviation	Bottom Quartile	Median	Upper Quartile
RRD	0.354	0.114	0.269	0.343	0.405
RRD with PVR	0.352	0.105	0.278	0.331	0.421
ERM	0.389	0.117	0.287	0.357	0.484
MH	0.375	0.151	0.278	0.375	0.433
Statistical analysis: F = 0.33, p = 0.81					

**Table 3:** Superoxide dismutase (SOD) activities (U/mg protein) in the studied groups (RRD—rhegmatogenous retinal detachment; PVR—proliferative vitreoretinopathy; ERM—epiretinal membrane; MH—macular hole).

Group	Average	Standard Deviation	Bottom Quartile	Median	Upper Quartile
RRD	10.505	5.686	7.000	9.275	13.340
RRD with PVR	11.697	6.192	6.330	9.960	15.070
ERM	11.501	3.498	9.360	9.750	14.200
MH	12.989	6.573	7.840	10.735	16.450
Statistical analysis: F = 0.53, p = 0.67					

**Table 4:** Glutathione reductase (GR) activities (U/g protein) in the studied groups (RRD—rhegmatogenous retinal detachment; PVR—proliferative vitreoretinopathy; ERM—epiretinal membrane; MH—macular hole).

Group	Average	Standard Deviation	Bottom Quartile	Median	Upper Quartile
RRD	3.560	1.722	2.297	3.171	5.103
RRD with PVR	3.792	1.473	2.699	3.764	4.791
ERM	3.601	1.217	2.956	3.570	3.848
MH	4.085	1.959	2.300	3.791	5.464
Statistical analysis: F = 0.33, p = 0.80					

**Table 5:** Disease duration (RRD—rhegmatogenous retinal detachment; PVR—proliferative vitreoretinopathy; ERM—epiretinal membrane; MH—macular hole) in days.

Group	Average	Standard Deviation	Bottom Quartile	Median	Upper Quartile
RRD	10.95	7.59	6.00	8.00	12.00
RRD with PVR	48.15	78.84	14.00	19.00	31.00
ERM	376.10	512.99	177.00	201.50	305.00
MH	258.57	74.66	207.00	247.00	307.00
Statistical analysis: H = 49.77, p < 0.0001					

**Table 6:** Correlation between the duration of the disease (RRD—rhegmatogenous retinal detachment; PVR—proliferative vitreoretinopathy) and antioxidant activity levels (TAS—total antioxidant status; SOD—superoxide dismutase; GR—glutathione reductase) in the groups.

Antioxidants	RRD with PVR		RRD	
	R	p	R	p
TAS	0.48	0.01	0.22	0.32
SOD	0.26	0.19	0.18	0.41
GR	0.18	0.36	0.10	0.65

## Data Availability

Data are available on reasonable request by the corresponding author.
